# Development and clinical validation of a novel six-gene signature for accurately predicting the recurrence risk of patients with stage II/III colorectal cancer

**DOI:** 10.1186/s12935-021-02070-z

**Published:** 2021-07-07

**Authors:** Zaoqu Liu, Taoyuan Lu, Jing Li, Libo Wang, Kaihao Xu, Qin Dang, Chunguang Guo, Long Liu, Dechao Jiao, Zhenqiang Sun, Xinwei Han

**Affiliations:** 1 Department of Interventional Radiology, The First Affiliated Hospital of Zhengzhou University, Zhengzhou, 450052 Henan China; 2 Interventional Institute of Zhengzhou University, Zhengzhou, 450052 Henan China; 3 Interventional Treatment and Clinical Research Center of Henan Province, Zhengzhou, 450052 Henan China; 4 Department of Cerebrovascular Disease, Zhengzhou University People’s Hospital, Zhengzhou, 450003 Henan China; 5 Department of Hepatobiliary and Pancreatic Surgery, The First Affiliated Hospital of Zhengzhou University, Zhengzhou, 450052 Henan China; 6 Department of Colorectal Surgery, The First Affiliated Hospital of Zhengzhou University, Zhengzhou, 450052 Henan China; 7 Department of Endovascular Surgery, The First Affiliated Hospital of Zhengzhou University, Zhengzhou, 450052 Henan China

**Keywords:** Stage II/III colorectal cancer, Recurrence, Gene signature, Adjuvant chemotherapy, LASSO

## Abstract

**Background:**

A large number of patients with stage II/III colorectal cancer (CRC) have a high recurrence rate after radical resection. We aimed to develop a novel tool to stratify patients with different recurrence-risk for optimizing decision-making in post-operative surveillance and therapeutic regimens.

**Methods:**

We retrospectively enrolled four independent cohorts from the Gene Expression Omnibus and 66 CRC tissues from our hospital. The initial signature discovery was conducted in GSE143985 (n = 91). This was followed by independent validation of this signature in GSE17536 (n = 111), GSE29621 (n = 40), and GSE92921 (n = 59). Further experimental validation using qRT-PCR assays (n = 66) was performed to ensure the robustness and clinical feasible of this signature.

**Results:**

We developed a novel recurrence-related signature consisting of six genes. This signature was validated to be significantly associated with dismal recurrence-free survival in five cohorts GSE143985 (HR: 4.296 [2.612–7.065], *P* < 0.0001), GSE17536 (HR: 2.354 [1.662–3.334], *P* < 0.0001), GSE29621 (HR: 3.934 [1.622–9.539], *P* = 0.0024), GSE92921 (HR: 7.080 [2.011–24.924], *P* = 0.0023), and qPCR assays (HR: 3.654 [2.217–6.020], *P* < 0.0001). This signature was also proven to be an independent recurrent factor. More importantly, this signature displayed excellent discrimination and calibration in predicting the recurrence-risk at 1–5 years, with most AUCs were above 0.9, average C-index for the five cohorts was 0.8795, and near-perfect calibration.

**Conclusions:**

We discovered and experimental validated a novel gene signature with stable and powerful performance for identifying patients at high recurrence-risk in stage II/III CRC.

**Supplementary Information:**

The online version contains supplementary material available at 10.1186/s12935-021-02070-z.

## Background

According to the latest global cancer statistics, colorectal cancer (CRC) remains the third most common cancer and second leading cause of cancer-associated death worldwide [1]. The current stage of CRC is classified based on the tumor-node-metastasis (TNM) system issued by the American Joint Committee on Cancer (AJCC), which is routinely standard for the prognostic management and determination of adjuvant chemotherapy (ACT) of early-stage CRC [2]. After surgery, ACT is a conventional therapy for stage III and a subset of stage II CRC patients (eg, high grade, T4) [3, 4]. The goal of ACT is to eradicate residual cancer cells after surgical resection, thus reducing the recurrence rate or extending the time to recurrence. However, a consider number of patients with stage II/III CRC relapse or suffer from drug side effects, and also, current guidelines declare that the present definition of “high-risk” stage II CRC is inadequate [5–7]. Previous studies have demonstrated that approximately half of stage III CRC patients will relapse within 5 years after surgical resection, while the 5-years recurrence rate of stage II CRC patients is about 12–38% [8–10]. In clinical practice, the AJCC stage system alone is limited due to patients within the same stage have heterogeneous clinical outcomes [11]. Therefore, it is essential to redefine the risk stratification for recurrence in order to identify stage II/III CRC patients who may truly require or can omit ACT.

Over the past few decades, intensive effort has been dedicated towards searching for new ways to evaluate the recurrence-risk of patients with early-stage CRC. We have previously demonstrated that the mutational status of *TTN*/*OBSCN* is an independent prognostic factor in CRC [12]. The mutation of *BRAF*, *KRAS*, and *PIK3CA*, loss of *SMAD4*, and amplification of *HER2*, are also reported to dramatically correlated with the recurrence of CRC [13–17]. Recently, Tie et al. have shown that circulating tumor DNA signature can serve as biomarkers of recurrence and benefit of ACT in stage III CRC [18]. In addition, the transcriptome-based consensus molecular subtype (CMS) and transcriptomic-based CRC intrinsic subtype (CRIS) systems have been reported to correlate with clinical outcomes in stage II/III CRC, and CMS4 or CRIS-C tumors have dismal recurrence and overall survival [19–21]. In parallel, patients with a low-frequency microsatellite instability (MSI-L) preferentially show a significantly increased risk of death and recurrence [22]. However, these classifiers only have a moderate prediction accuracy and limited clinical usefulness [23, 24].

Notably, an immunohistochemistry-based scoring pipeline has been established and validated (termed Immunoscore^®^), which quantifies the densities of two adaptive immune cells, CD3 + and CD8 + T cells, in the core and invasive margin of tumor [24]. Although Immunoscore^®^ displays a stable predictive power of prognosis in early-stage CRC, its performance remains at a moderate accuracy of Harrell’s C-statistics ranging from 0.56 to 0.68 in international researches [24]. This may be due to the fact that only two adaptive immune cells are considered, but other components in tumor may also be vital for evaluating recurrence-risk of early CRC. Our hypothesis is that comprehensive identification of key recurrence-associated genes and construction of predictive model will improve the accuracy of recurrence-risk assessment in stage II/III CRC. Traditional techniques such as immunohistochemistry or quantitative real-time polymerase chain reaction (qRT-PCR) are inconvenient to quantify a remarkable number of genes, but advances in bioinformatics have made it possible. Nowadays, we can easily obtain a large scale of genes for downstream analysis. With the help of machine learning, such as the least absolute shrinkage and selection operator (LASSO) algorithm, it is possible to identify the most important elements based on the expression profiles of global genes and fit a model with strong generalization performance [25].

In the present study, using four independent public cohorts with gene expression and recurrence-free survival (RFS) data, we performed a systematic and comprehensive biomarker discovery and validation work to develop a CRC recurrence-risk score (CRRS) system for predicting the RFS of patients with stage II/III CRC. Furthermore, we used 66 frozen tissue samples with qRT-PCR data for experimental verification to prove the stability and reliability of CRRS. Herein, we report a novel six-gene signature, which not only offers stable and excellent accuracy in identifying patients at high recurrence-risk, but also can be readily translated into the clinical practice due to the simplicity and inexpensiveness of PCR-based assays. Overall, we believe CRRS offers an attractive platform for evaluating recurrence-risk of patients with stage II/III CRC, and has important significance in optimizing decision-making in post-operative surveillance and therapeutic regimens.

## Methods

### Public data collection and processing

Four retrospective CRC cohorts were enrolled from Gene Expression Omnibus (GEO, http://www.ncbi.nlm.nih.gov/geo) database, including GSE143985, GSE17536, GSE29621, and GSE92921. These cohorts all belong to the Affymetrix^®^ GPL570 platform ([HG-U133 Plus 2] Affymetrix^®^ Human Genome U133 Plus 2.0 Array). The raw data from Affymetrix^®^ were processing using the robust multiarray averaging (RMA) algorithm implemented in the affy R package. RMA was used to perform background adjustment, quantile normalization, and final summarization of oligonucleotides per transcript using the median polish algorithm. In four cohorts, we only retained CRC patients that met the following criteria: (1) Primary tumor tissues samples; (2) In the AJCC stage II/III; (3) Have both recurrent status and RFS information; (4) No preoperative chemotherapy or radiotherapy received. A total of 91 patients from GSE143985 were used as the training set, and GSE17536 (n = 111), GSE29621 (n = 40), and GSE92921 (n = 59) were used as the validation sets. The baseline data were summarized in Additional file 1: Table S1. The time from surgery to cancer recurrence was defined as RFS.

### Signature generation

First, based on univariate Cox regression, genes with *P* < 0.05 and hazard ratio (HR) consistently > 1 or < 1 in all enrolled cohorts were defined as stable recurrence-associated genes. Next, using the expression of these stable recurrence-associated genes in training set, we developed a CRC recurrence-risk score (CRRS) system to predict the RFS of patients with stage II/III CRC via the LASSO Cox regression algorithm. By ten-fold cross validation, the optimal lambda was generated when the partial likelihood deviance reached the minimum value. Finally, based on the optimal lambda, genes with nonzero coefficients were selected to establish the prediction model. The CRRS for each patient was calculated with the LASSO model weighting coefficient as follows:

$$CRRS{\text{ = }}\sum\limits_{{i = 1}}^{n} {Exp_{i} \times Coef_{i} }$$

where *n* is the number of key genes, *Exp*_*i*_ is the expression of gene *i*, and *Coef*_*i*_ is the LASSO coefficient of gene *i*.

### Human tissue specimens and qRT-PCR analysis

From January 2015 to December 2016, we collected a total of 66 frozen surgically resected CRC tissues with AJCC stage II/III at The First Affiliated Hospital of Zhengzhou University. Follow up was concluded five years after surgery. Detailed baseline data of CRC patients were displayed in Additional file 1: Table S1. Total RNA was isolated from CRC tissues using RNAiso Plus reagent (Takara, Dalian, China) according to the manufacturer’s instructions. RNA quality was evaluated using a NanoDrop One C (Waltham, MA, USA), and RNA integrity was assessed using agarose gel electrophoresis. An aliquot of 1 µg of total RNA was reverse-transcribed into complementary DNA (cDNA) according to the manufacturer’s protocol using the miRNA reverse transcription Kit (TaKaRa BIO, Japan). All cDNA samples were prepared for qRT-PCR. This project was approved by the Ethics Committee Board of The First Affiliated Hospital of Zhengzhou University. In the qRT-PCR analysis, the enrolled 6 genes in the model were detected. qRT-PCR was performed using SYBR Assay I Low ROX (Eurogentec, USA) and SYBR^®^ Green PCR Master Mix (Yeason, Shanghai, China). The expression value of the target genes was normalized to *GAPDH*, and then log2 transformed for subsequent analysis. The primer sequences of the included 6 genes and *GAPDH* were shown in Additional file 1: Table S2.

### Statistical analysis

All data processing, statistical analysis, and plotting were conducted in R 4.0.3 software. Continuous variables were compared between two groups through the Wilcoxon rank-sum test. Fisher’s exact test was applied to compare categorical variables. The patients were divided into high and low-risk groups based on the median risk score. The Kaplan–Meier method and the log-rank test were utilized to estimate the different RFS between two groups. The receiver operating characteristic curve (ROC) used to predict binary categorical variables was implemented using the R package pROC. The time-dependent area under the ROC (AUC) for survival variable were conducted by the R package timeROC. The R package rms was applied to plot calibration curves. All statistical tests were two-sided. *P* < 0.05 was regarded as statistically significant.

## Results

### Baseline characteristics of patients

As displayed in Additional file 1: Table S1, we enrolled a total of 367 patients with stage II/III CRC from five independent multicenter cohorts. In GSE143985, there were 55 stage II and 36 stage III patients with a median RFS of 5.8904 years; the 1-, 2-, and 3-year recurrence rate were 9.9%, 12.1%, and 15.4%, respectively. In GSE17536, there were 55 stage II and 56 stage III patients with a median RFS of 3.0625 years; the 1-, 2-, and 3-year recurrence rate were 6.3%, 15.3%, and 23.4%, respectively. In GSE29621, there were 22 stage II and 18 stage III patients with a median RFS of 3.9379 years; the 1-, 2-, and 3-year recurrence rate were 7.5%, 10.0%, and 15.0%, respectively. In GSE92921, there were 43 stage II and 16 stage III patients with a median RFS of 5.7260 years; the 1-, 2-, and 3-year recurrence rate were 6.8%, 8.5%, and 10.2%, respectively. In qRT-PCR validation cohort, there were 40 stage II and 26 stage III patients with a median RFS of 3.9671 years; the 1-, 2-, and 3-year recurrence rate were 15.2%, 16.7%, and 21.2%, respectively.

### Establishment and validation of CRRS with stage II/III CRC in public datasets

**Fig. 1:**
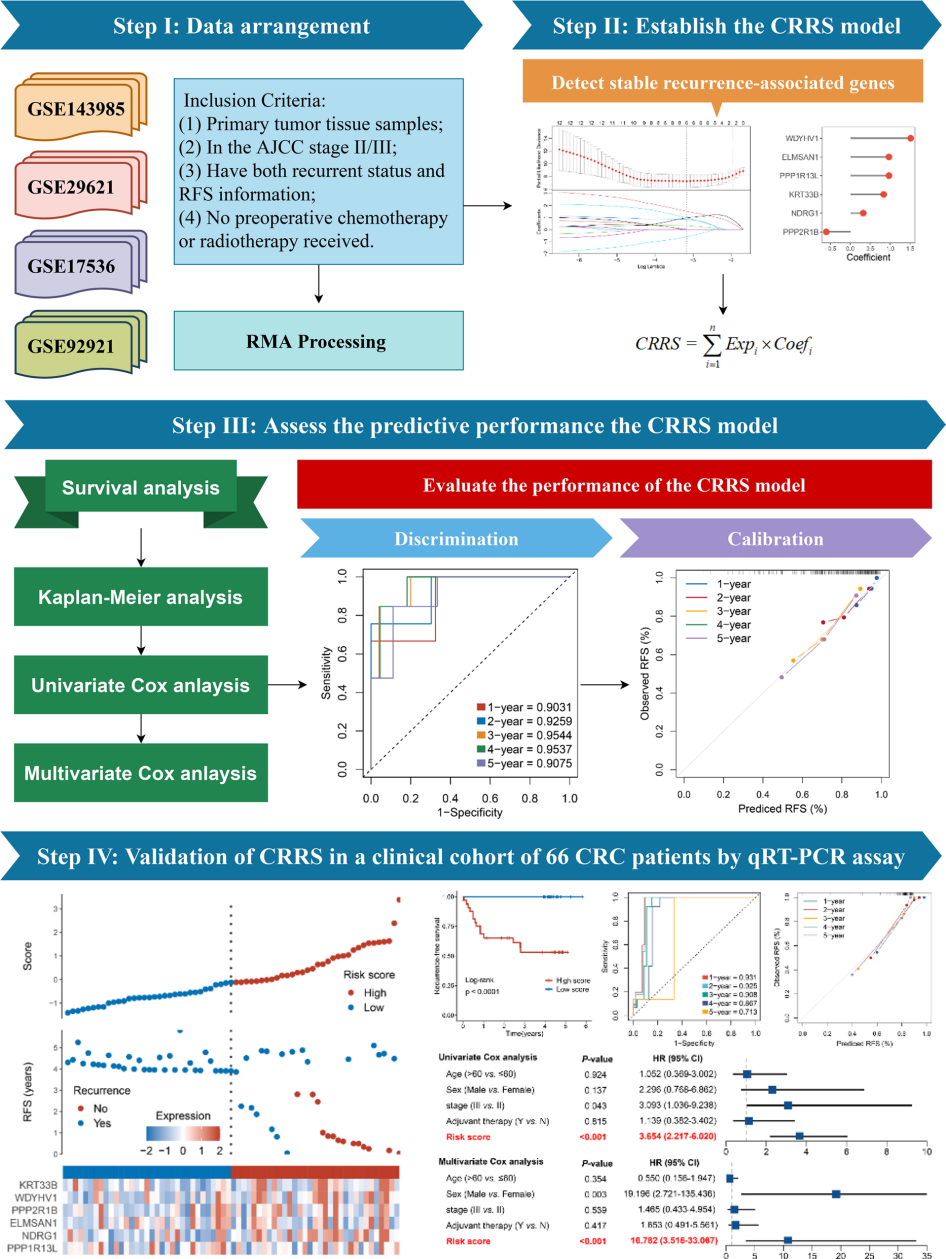
The flowchart of this study

**Fig. 2:**
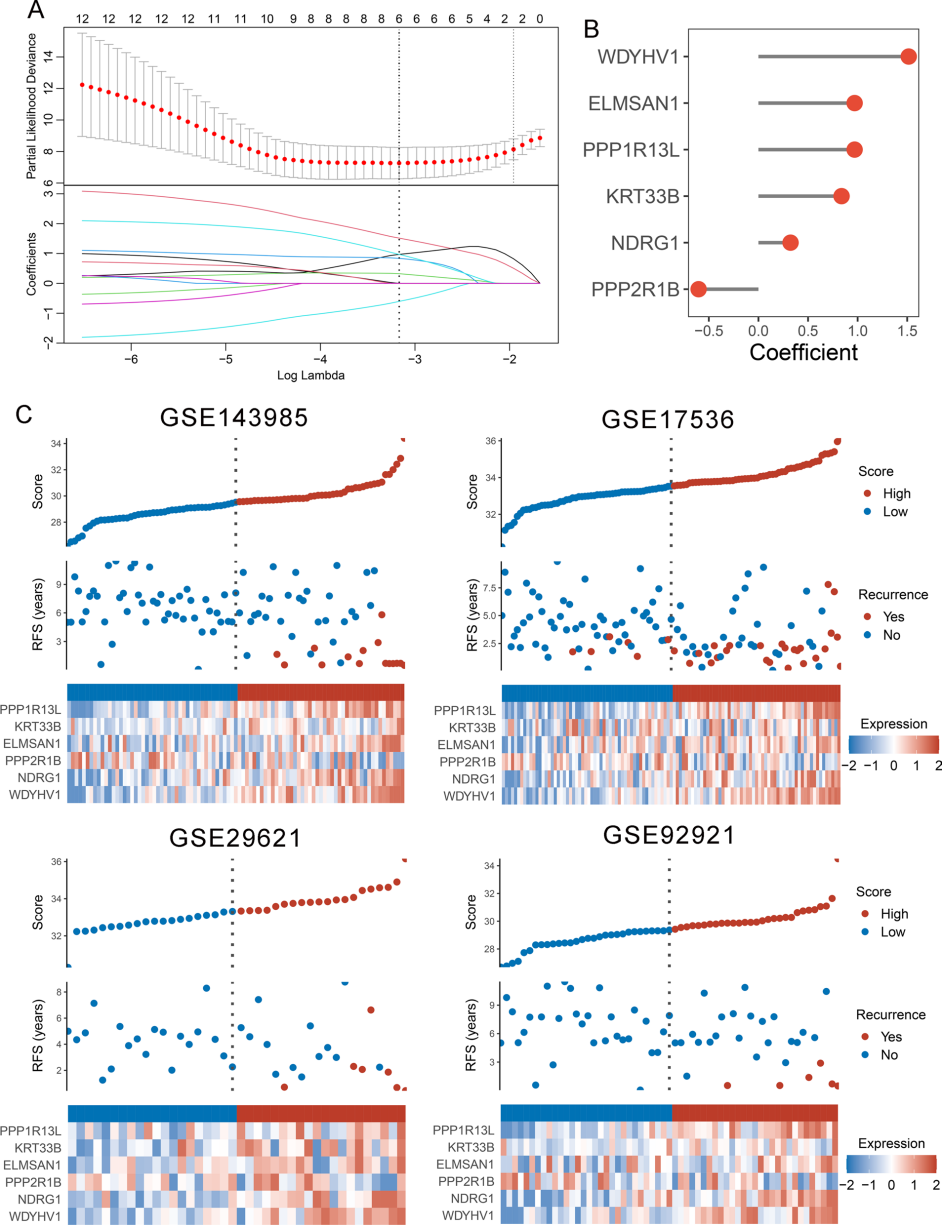
The development of the CRRS model based on the LASSO algorithm. **A** Ten-fold cross-validations to tune the parameter selection in the LASSO model. The two dotted vertical lines are drawn at the optimal values by minimum criteria (left) and 1 − SE (standard error) criteria (right). **B** LASSO coefficient profiles of the candidate genes for CRRS construction. **C** The distribution of risk score, recurrence status, and gene expression panel in four cohort

**Fig. 3:**
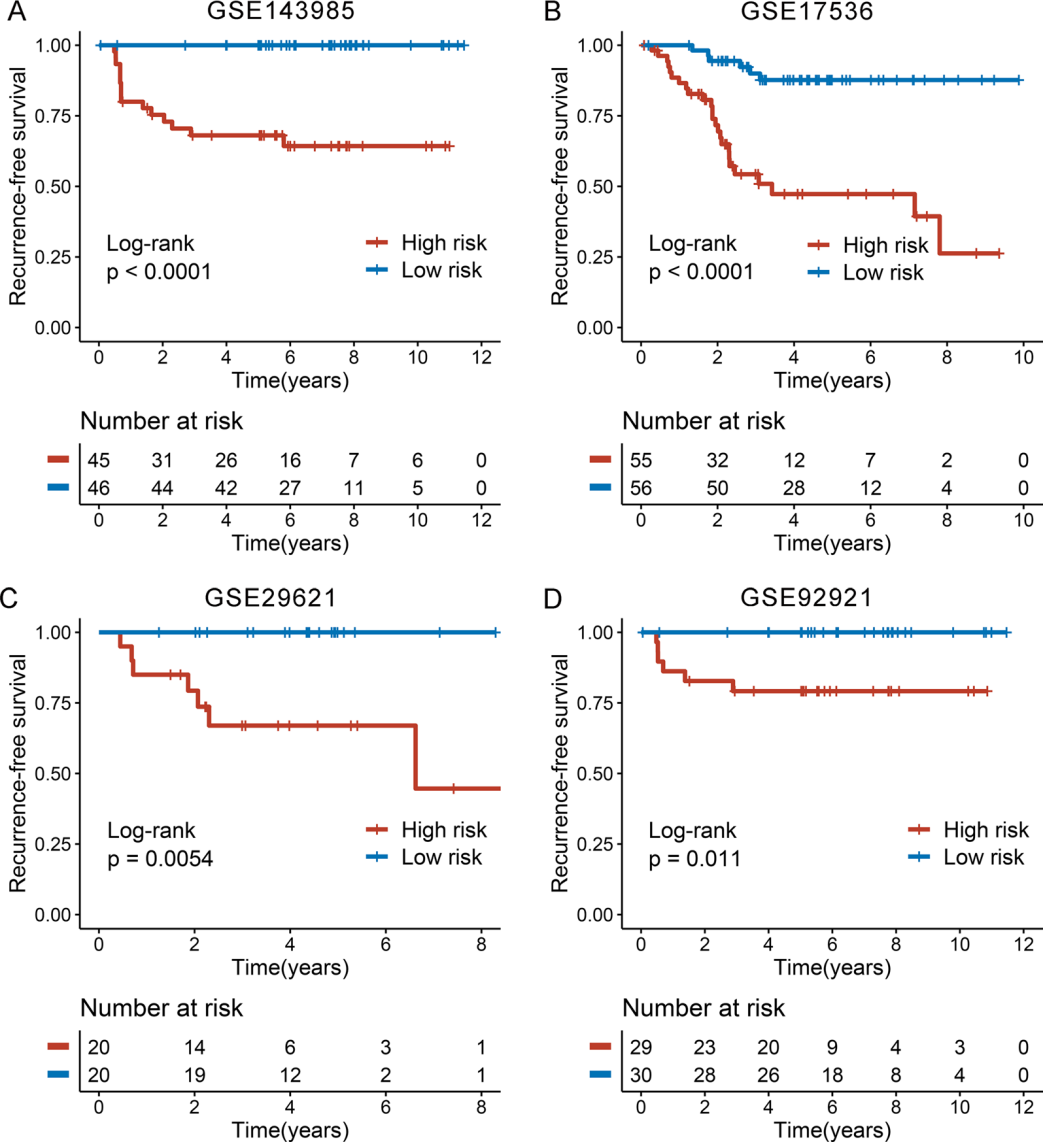
Kaplan–Meier survival analysis of CRRS in four cohorts. Kaplan–Meier curves of RFS according to the CRRS in GSE143985 (**A**), GSE17536 (**B**), GSE29621 (**C**), and GSE92921 (**D**)

The workflow was shown in Fig. 1. In total, univariate Cox results of four cohorts identified 13 genes were stably associated with RFS (all *P* < 0.05) (Additional file 1: Table S3). Based on the expression of these genes in GSE143985, we fitted a LASSO Cox regression model and identified 6 genes that were strongly predictive of RFS, encompassing *ELMSAN1, KRT33B, NDRG1, PPP1R13L, PPP2R1B,* and *WDYHV1* (Fig. 2A). Next, the CRRS was calculated using a formula that including the 6 genes weighted by their regression coefficients in a penalized Cox model as follows: CRRS = 0.9698 × Exp (*ELMSAN1*) + 0.8381 × Exp (*KRT33B*) + 0.3238 × Exp (*NDRG1*) + 0.9697 × Exp (*PPP1R13L*)—0.6033 × Exp (*PPP2R1B*) + 1.5152 × Exp (*WDYHV1*) (Fig. 2B). The CRRS of each patient calculated according to this formula. Expression heatmap of the 6 selected genes, distribution of CRRS, and recurrent status of each patient were illustrated in Fig. 2C. In four cohorts, all patients were segmented into high- and low-risk groups based on the median CRRS (Fig. 2C). Compared with the low-risk group, patients in the high-risk group displayed significantly unfavorable RFS in GSE143985 (HR: 4.296 [2.612–7.065], log-rank *P* < 0.0001; Fig. 3A), GSE17536 (HR: 2.354 [1.662–3.334], log-rank *P* < 0.0001; Fig. 3B), GSE29621 (HR: 3.934 [1.622–9.539], log-rank *P* = 0.0054; Fig. 3C), and GSE92921 (HR: 7.080 [2.011–24.924], log-rank *P* = 0.0110; Fig. 3D) (Table 1). After adjusting the available clinical characteristics in four cohorts, multivariate Cox regression analysis revealed CRRS remained an independent risk factor for evaluating RFS of stage II/III CRC patients (all *P* < 0.01) (Table 1).

**Table 1:** Univariate and multivariate Cox regression analysis of the risk score

Characteristics	Univariate Cox analysis		Multivariate Cox analysis	
	HR (95%CI)	*P*-value	HR (95%CI)	*P*-value
GSE143985				
Stage (III vs II)	4.058 (1.272–12.943)	0.018	6.876 (1.276–37.041)	0.025
ACT (Y vs N)	2.144 (0.743–6.186)	0.158	0.989 (0.210–4.649)	0.989
TP53 (Mut vs Wt)	0.400 (0.134–1.195)	0.101	1.058 (0.270–4.152)	0.935
KRAS (Mut vs Wt)	3.182 (1.065–9.510)	0.038	2.289 (0.571–9.185)	0.243
Risk score	4.296 (2.612–7.065)	< 0.001	5.128 (2.662–9.880)	< 0.001
GSE17536				
Stage (III vs II)	1.964 (0.941–4.099)	0.072	0.967 (0.402–2.326)	0.142
Age (> 60 vs ≤ 60)	0.482 (0.238–0.978)	0.043	0.728 (0.308–1.721)	0.009
Sex (Male vs Female)	1.138 (0.562–2.304)	0.720	3.155 (1.196–8.321)	0.370
Risk score	2.354 (1.662–3.334)	< 0.001	2.527 (1.720–3.705)	< 0.001
GSE29621				
Stage (III vs II)	2.785 (0.525–14.774)	0.229	0.886 (0.093–8.072)	0.900
Age (> 60 vs ≤ 60)	1.083 (0.242–4.849)	0.917	0.888 (0.095–8.253)	0.917
ACT (Y vs N)	1.628 (0.311–8.531)	0.564	5.698 (0.323–100.67)	0.235
Risk score	3.934 (1.622–9.539)	0.002	5.150 (1.558–17.030)	0.007
GSE92921				
Stage (III vs II)	6.229 (1.140–34.035)	0.035	3.706 (0.585–23.486)	0.164
TP53 (Mut vs Wt)	1.453 (0.266–7.932)	0.666	1.050 (0.165–6.677)	0.959
KRAS (Mut vs Wt)	3.217 (0.589–17.580)	0.177	2.720 (0.280–26.448)	0.389
Risk score	7.080 (2.011–24.924)	0.002	6.311 (1.691–23.562)	0.006

### Predictive performance the CRRS model

**Fig. 4:**
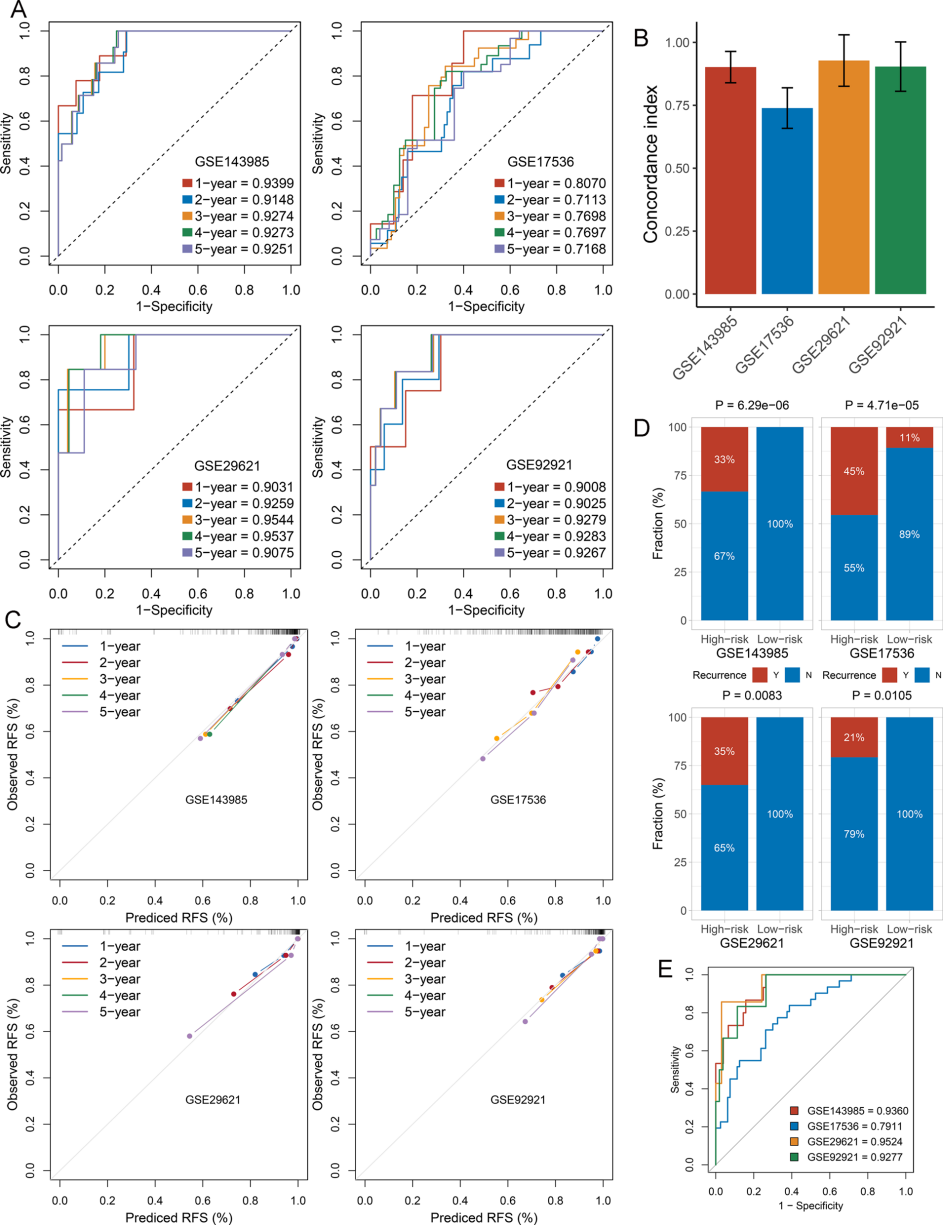
Evaluation of the CRRS model in four cohorts. **A** Time-dependent ROC analysis for predicting RFS at 1 ~ 5 years. **B** The Harrell’s C-index of CRRS. **C** Calibration plots for comparing the actual probabilities and the predicted probabilities of RFS at 1 ~ 5 years. **D** Comparison of recurrence rate between the high-risk and low-risk groups. **E** ROC analysis of the CRRS model for predicting the recurrence event of patients

In this study, ROC and concordance index (C-index) as well as calibration curve were utilized to evaluate the discrimination and calibration of CRRS, respectively. The results showed that the AUCs for predicting RFS at 1–5 years was 0.9399, 0.9148, 0.9274, 0.9273, and 0.9251 in GSE143985, 0.8070, 0.7113, 0.7698, 0.7697, and 0.7168 in GSE17536, 0.9031, 0.9259, 0.9544, 0.9537, and 0.9075 in GSE29621, and 0.9008, 0.9025, 0.9279, 0.9283, and 0.9267 in GSE92921, respectively (Fig. 4A). The C-index were 0.9018 [0.8396–0.9640], 0.7390 [0.6584–0.8196], 0.9279 [0.8256–1], and 0.9037 [0.8057–1] in four cohorts, respectively (Fig. 4B). These results suggested this model possessed high predative accuracy for predicting recurrence-risk at 1–5 years. Moreover, the CRRS showed excellent calibration, with the predicted probabilities of RFS at 1–5 years accurately, describing the true risk observed in all four cohorts (Fig. 4C). The CRRS also accurately separated the recurrence and recurrence-free CRC with tumor stage II/III after surgical resection. As illustrated in Fig. 4D, patients in the high-risk group displayed a significantly higher fraction of recurrence (high-risk *vs*. low-risk: 33% *vs*. 0% in GSE143985, 45% *vs*. 11% in GSE17536, 35% *vs*. 0% in GSE29621, and 21% *vs*. 0% in GSE92921; all *P* < 0.05). It can be observed that CRRS perfectly separated recurrence and recurrence-free CRC in three cohorts. The ROC analysis further suggested the CRRS possessed high accuracy for identifying CRC patients with recurrence in all four cohorts (AUC: GSE143985 = 0.9360, GSE17536 = 0.7911, GSE29621 = 0.9524, GSE92921 = 0.9277) (Fig. 4E). Overall, in four public cohorts, the CRRS presented stable and excellent performance in evaluating RFS in patients with stage II/III CRC after surgical resection.

### Validation of CRRS in a clinical in-house cohort

**Fig. 5:**
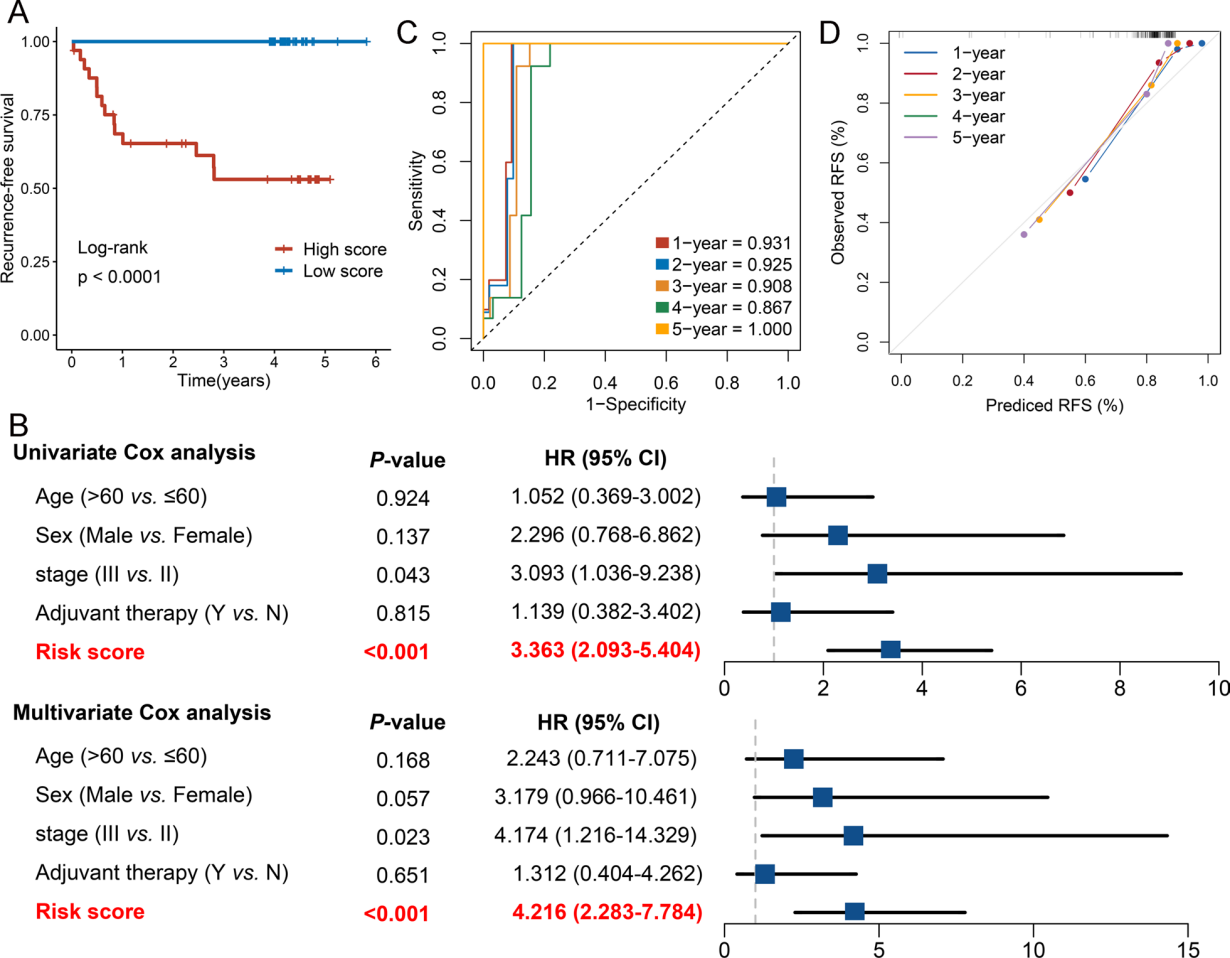
Validation of our discovery in a clinical in-house cohort. **A** Kaplan–Meier curves of RFS according to the CRRS. **B** Univariate and multivariate Cox regression analysis of the risk score. **C** Time-dependent ROC analysis for predicting RFS at 1–5 years. **D** Calibration plots for comparing the actual probabilities and the predicted probabilities of RFS at 1–5 years

In order to verify the power of our six-gene CRRS model into a clinically translatable risk-stratification assay, we further performed qRT-PCR assays for these genes in a clinical cohort containing 66 CRC patients. Expression heatmap of the 6 selected genes, distribution of CRRS, and recurrent status of each patient were illustrated in Additional file 2: Fig. S1A. In line with our discovery in-silico validation cohorts, patients with high score have the significantly dismal RFS (HR: 3.363 [2.093–5.404], log-rank *P* < 0.0001; Fig. 5A, B). Multivariate Cox regression analysis revealed that the CRRS remained the statistical significance (HR: 4.216 [2.283–7.784], *P* < 0.0001; Fig. 5B), after adjusting for potential confounding factors (including age, sex, stage, and ACT). The time-dependent ROC analysis showed the pinpoint accuracy of CRRS: the AUCs for predicting RFS at 1 ~ 5 years was 0.931, 0.925, 0.908, 0.867, and 1.000, respectively (Fig. 5C). Likewise, the C-index reached 0.925 [0.862–0.988]. The calibration plot further displayed the predicted probabilities of RFS at 1–5 years accurately describing the true risk observed (Fig. 5D). The CRRS model perfectly distinguished recurrent CRC from non-recurrent CRC (high-risk *vs*. low-risk: 42% *vs*. 0%; Additional file 2: Fig. S1B), with a high precision AUC = 0.919 (Additional file 2: Fig. S1C). Of note, CRRS also showed a significantly higher accuracy than age, gender, stage, and chemotherapy (Additional file 2: Fig. S1D). Collectively, the results from a clinical in-house cohort supported that our discovery and in-silico validation cohort findings, which validated and confirmed that our CRRS model was quite robust, and can serve as an independent predictor of recurrence in stage II/III CRC.

## Discussion

CRC is a highly heterogeneous tumor with complex biological processes and molecular mechanism, for which post-operative surveillance and therapeutic regimens are necessary to be tailored to generate an optimal outcome for each patient. Nevertheless, a considerable proportion of stage II/III CRC patients not only derive benefit from 5-Fluorouracil (5-FU)-based adjuvant chemotherapy but also display drug reactions [5, 6]. A limitation of the current AJCC stage system is that patients in the same stage have distinct clinical outcomes, which leads to latent under- or over-treatment. Therefore, developing a novel classifier that can be routinely implemented into clinical practice is critical for identifying those early-stage patients who are at high recurrence-risk and who might thus benefit from adjuvant chemotherapy. We hypothesized that a signature with high performance could be developed according to the global immune milieu. With the development of artificial intelligence and bioinformatics, an advanced machine learning algorithm can identify several key indicators that are most meaningful to predict clinical outcomes from a large number of genes [25], which is actually in line with the biological scale-free network which was dominated by a few hub nodes [26]. Therefore, for the first time, we developed a novel signature (termed CRRS) to evaluate recurrence-risk of patients with stage II/III CRC in multicenter cohorts. The reproducibility and powerful performance of CRRS in multiple independent cohorts and external qRT-PCR data not only prove that it is a robust and highly accurate model, but also is promising to be routinely implemented into clinical practice due to the advantages of high sensitivity and specificity, simplicity, and low cost of qRT-PCR.

In this study, we fitted a recurrence model consisting of 6 genes, including *ELMSAN1, KRT33B, NDRG1, PPP1R13L, PPP2R1B,* and *WDYHV1* [27–31]. A majority of genes have been reported to be involved in the initiation and progression of tumor. For example, the downregulation of NDRG1 is associated with tumor metastasis via inducing epithelial-mesenchymal transition [28]; the expression of *PPP2R1B* was regulated by miRNA-587 to antagonizes 5-FU-induced apoptosis in CRC [30]. Of note, the role of *ELMSAN1* has not previously been reported in cancer, and thus requires further exploration. Based on the 6 enrolled genes, we developed the CRRS model, which performed stably in predicting recurrent-risk of patients with stage II/III CRC. The prognostic meta-analysis showed that RAIS was a risk indicator of recurrence and was proven to be an independent factor after adjusting multiple clinical clinicopathologic features. More importantly, in four cohorts, CRRS demonstrated a high discrimination and calibration in predicting the recurrence-risk at 1–5 years. To prevent false positive results from sequencing data, we conducted another validation according to qRT-PCR results from 66 frozen CRC tissues with tumor stage II/III, confirming our prior findings and evaluating their practicality in different centers. As reported previously, patients with a high-risk score suggested dismal RFS, and thus might need to adjust therapy strategies or add additional adjuvant chemotherapy. For example, current guidelines recommend that a subset of stage II patients without “high-risk” traits do not require adjuvant chemotherapy [7], but when these patients show a high-risk score, using additional adjuvant chemotherapy might be essential.

Prior to this study, a few reports established molecular signatures for predicting prognostic risk of CRC [32–36]. In comparison with these studies, our work has several advantages and novelties: (1) The CRRS model was developed based on the recurrence rather than overall survival in patients with stage II/III CRC, which allowed it to accurately identify high-risk patients with early-stage CRC; (2) Fewer genes comprising the signature makes the CRRS easier to implement; (3) We performed comprehensive statistical approaches to evaluate the discrimination and calibration of the CRRS model, and our model remained stable and highly accurate performance at 1–5 years; (4) qRT-PCR was used to validate the performance of CRRS to ensure its robustness and clinical feasible. Despite the CRRS model is promising, some limitations should be acknowledged. First, all the samples from five centers were retrospective, and future validation of the CRRS model should be conducted in prospective fresh samples. Second, some clinical characteristics on public datasets were very inadequate, which thus had concealed the potential associations between CRRS and some clinical traits.

## Conclusions

In summary, using a systematic and comprehensive biomarker discovery and validation approach, we established and validated a stable and powerful six-gene signature for evaluating the recurrence-risk of patients with stage II/III CRC. Our study demonstrated the CRRS model may be a promising tool to optimize decision-making in surveillance protocol and ACT for individual patients with stage II/III CRC.

## Supplementary Information

**Additional file 1: Table S1.** Details of baseline information in GSE143985, GSE17536, GSE29621, GSE92921 and qRT-PCR data from 66 samples, respectively. **Table S2.** The forward and reverse primers for qRT-PCR. **Table S3.** Univariate Cox results of four cohorts revealed a total of 13 stable genes (red mark) were significantly associated with RFS.

**Additional file 2: Fig. S1.** Validation of CRRS in a clinical in-house cohort. **A.** The distribution of risk score, recurrence status, and gene expression panel in four cohort. **B.** Comparison of recurrence rate between the high-risk and low-risk groups. **C.** ROC analysis of the CRRS model for predicting the recurrence event of patients. **D**. C-index of CRRS, age, gender, stage, and chemotherapy for evaluating recurrence-free survival.

## Data Availability

Public data used in this work can be acquired from the TCGA Research Network portal (https://portal.gdc.cancer.gov/) and Gene Expression Omnibus (GEO, http://www.ncbi.nlm.nih.gov/geo/).

## Abbreviations

CRC: Colorectal cancer
TNM: Tumor-node-metastasis
AJCC: American Joint Committee on Cancer
ACT: Adjuvant chemotherapy
CMS: Consensus molecular subtype
CRIS: Intrinsic subtype
MSI-L: Low-frequency microsatellite instability
qRT-PCR: Quantitative real-time polymerase chain reaction
LASSO: Least absolute shrinkage and selection operator
RFS: Recurrence-free survival
CRRS: CRC recurrence-risk score
ROC: Receiver operating characteristic curve
AUC: Area under the ROC
C-index: Concordance index
