# Supplementary figures and images for: Development and clinical validation of a novel six-gene signature for accurately predicting the recurrence risk of patients with stage II/III colorectal cancer
Source: Cancer Cell Int. 2021 Jul 7;21:359. doi: 10.1186/s12935-021-02070-z (PMC8265123; doi:10.1186/s12935-021-02070-z)

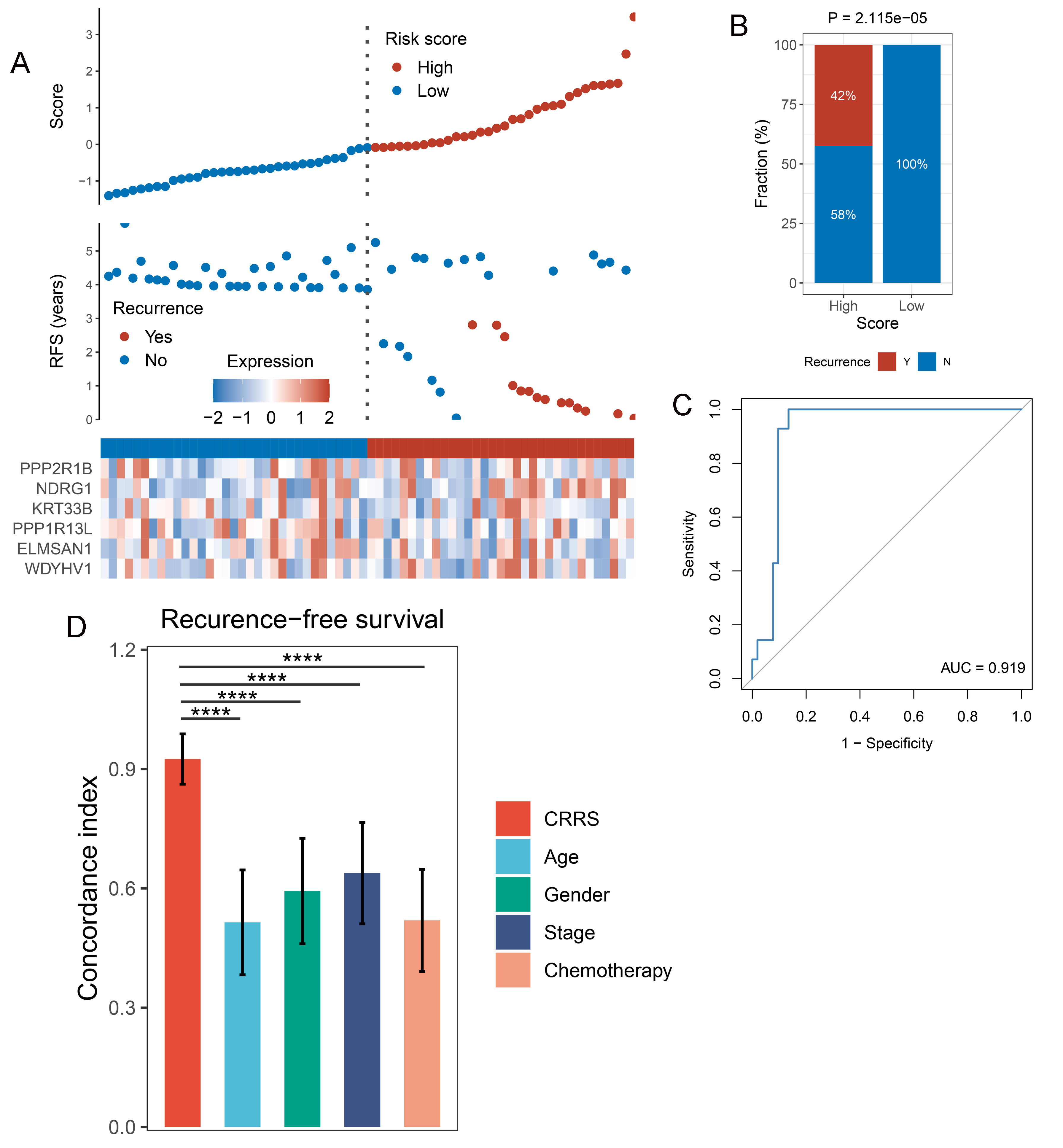

Supplement: Supplementary file 2 — Additional file 2: Fig. S1. Validation of CRRS in a clinical in-house cohort. A. The distribution of risk score, recurrence status, and gene expression panel in four cohort. B. Comparison of recurrence rate between the high-risk and low-risk groups. C. ROC analysis of the CRRS model for predicting the recurrence event of patients. D. C-index of CRRS, age, gender, stage, and chemotherapy for evaluating recurrence-free survival. [file 12935_2021_2070_MOESM2_ESM.tif]
